# Social Perceptions of Gender Differences and the Subjective Significance of the Gender Inequality Issue

**DOI:** 10.11621/pir.2022.0205

**Published:** 2022-06-30

**Authors:** Svetlana D. Gurieva, Tatiana V. Kazantseva, Larisa V. Mararitsa, Olga E. Gundelakh

**Affiliations:** a Saint Petersburg State University, Saint Petersburg, Russia

**Keywords:** Gender differences, gender inequality, social perceptions of gender differences, traditionalism, egalitarianism

## Abstract

**Background:**

Gender inequality continues to reproduce itself in hidden and ambivalent forms and leads to invisible barriers in women’s careers and lives. The authors were interested in how social perceptions of gender differences would relate to the maintenance of gender inequality in various spheres of life.

**Objective:**

The purpose of the presented research was to study social perceptions of gender differences in relation to the subjective significance of the gender inequality issue.

**Design:**

The study was conducted via an online survey throughout February-September of 2019. The sample included 106 people aged 18 to 68 (M = 30.2, σ = 10.5), 49% of respondents were women. The authors have developed and tested a questionnaire assessing the adherence to ideas regarding evident gender differences in various spheres of life. The reliability of all scales of the questionnaire has been tested. Respondents also completed a questionnaire identifying their perceptions of gender inequality and shared their life experience with respect to this phenomenon in the form of free description.

**Results:**

The following two latent factors reflecting different aspects of gender perceptions have been identified: “Career Inequality” and “Differences in Social Spheres”. Indicators of the subjective significance of gender inequality (which include gender awareness, frequency of gender inequality witnessing, personal experience of gender discrimination and the emotional significance of this experience) were positively correlated with perceptions of career inequalities (these support ideas regarding gender differences when it comes to opportunities for professional realization) and negatively correlated with perceptions of differences within social spheres (these support ideas regarding the existence of essential gender differences within the family, politics and everyday life).

**Conclusion:**

Articulation of personal experiences of gender inequality is associated with social perceptions of the absence of essential gender differences in various social domains (egalitarianism) and sensitivity to gender inequality with regards to career opportunities.

## Introduction

The problem of perception of gender differences and, as a result, gender inequality remains a topical issue today. Gender inequality in society, organizations, business and politics continues to exist despite the fact that the nature of gender inequality and social circumstances have changed. The persistence of gender inequality has been called a phenomenon of “stalled progress” (Cohen, Huffman, & Knauer, 2009). An example of this according to Andresen, Biemann and Pattie (2015) is the unceasing inequality between men and women as far as status and income are concerned, despite the significant increase in the share of women participating in the economy. There is an increased interest in gender differences due to the rapid growth of women’s wealth and the resulting economic behavior. Studies (Charness & Gneezy, 2012; 2018) have confirmed that women subjectively perceive themselves as less financially literate than men and, therefore, trade less frequently on stock exchanges (Charness & Gneezy, 2012) and invest with less risk compared to men (Barber & Odean, 2001). Similarly, behavioral differences between genders were found in other social domains, such as communication and negotiation (Mazei et al., 2015), networking (Forret & Dougherty, 2004) and parenting (Yaffe, 2020), commonly claiming more self-assertive and dominating behaviors in men. The data show that these behavioral differences are linked by several cognitive phenomena inhibiting women’s success, such as fear of success, attribution of failure as a lack of abilities, a decrease in self-efficacy (Mednick & Thomas, 2008) as well as the stereotype threat effect — poorer performance out of fear of fulfilling a negative stereotype (Nelson, 2009).

The socio-psychological view of the problem of gender involves the study of social representations of gender differences, gender stereotypes and their influence on different spheres of human life. Stereotypes become social norms, prescribing appropriate behavior for men and women, and simultaneously transforming gender differences into gender inequality.

Research indicates that stereotypes, as a derivative of the social context and social structures, influence the emergence and maintenance of sexism and gender inequality (Morgenroth & Ryan, 2018; Stewart et al., 2021). Human society has done a lot to reduce the gender gap in healthcare and education, but it is still far from establishing equal income for women and men (Shawn & Glenn, 2010). Psychologists describe several mechanisms of discrimination persistence. Firstly, according to gender-role theory (Eagly & Wood, 1991), women and men are represented differently in different social roles. The biological ability of women to bear children leads to the perception of them as vulnerable, weak and in need of protection and, as a result, men are perceived as strong and responsible for them (Hollander, 2001; Koening, 2018). Secondly, the nature of intergroup relations, that is, relations between men and women as social groups, plays a role. Relations between any groups are characterized by two basic criteria: the distribution of power and the valence of the relationship (hostility or benevolence). According to the theory of ambivalent sexism (Glick & Fiske, 2001; Connor, Glick & Fiske, 2017), gender discrimination contains both negative and positive prejudices against men and women. Women, for example, may be perceived as requiring nurturing and patronage (a phenomenon of “protective paternalism”) (Salomon et al., 2020) and this fact has both desirable and undesirable consequences for women, because it essentially demonstrates gender inequality.

However, most socio-psychological models emphasize how gender-specific behavioral patterns emerge, are shaped, and supported (*Table 1*). Thus, for example, biological models (Hutt, 1972; Wilson, 2000) argue that genetic, hormonal, and physiological factors influence gender differences; meanwhile psychoanalytic theory, the theory of social learning and the theory of cognitive development suggest that early learning fully defines the differences in adult gendered behavior. Sociological models such as social role theory (Eagly & Sczesny, 2019; Eagly & Wood, 1991; Thang, 2002) and expectation states theory (Berger, Rosenholtz, & Zelditch, 1980) suggest that specific aspects of social structure, such as the distribution of women and men into different social roles, contribute to persistent behavioral differences between women and men. The existence of gender stereotypes and restrictions for women in the professional sphere has been described in many works (Abraham, 2020; Acker, 1990; 2006; Benschop, 2009; Cohen et al., 2009; Coleman, 1988; Gurieva et al., 2016; Gurieva & Udavikhina, 2015). The main manifestations of gender segregation in employment are the traditional divisions of professions into “female” and “male” ones, the difference in wages and unemployment rates. The theoretical perspective takes a cross-cultural approach, claiming that gender behavior is conditioned by cultural and historical context (Lytton & Romney, 1991; Rudman & Fairchild, 2004; Rudman & Phelan, 2008; Vandello & Bosson, 2013).

**Table 1:** Theoretical approaches to studying gender behavior

Theoretical approaches	Content	Sources
Biological approaches.	Arguments for genetic, hormonal, and physiological factors of behav- ior.	Hutt (1972); Wilson (2000).
The theory of social learning, the theory of cognitive development and psychoanalytic theory.	Early learning fully defines the dif- ferences in adult gendered behavior.	Maccoby (1990); Maccoby & Jacklin (1987).
Sociological/ socio-psychological theories: The expectation states theory and the social roles theory.	Aspects of social structure, the distribution of women and men in different social roles, contribute to sustained behavioral differences.	Berger et al. (1980); Eagly & Wood (1991).
The cross-cultural approach.	Gender behavior is shaped and anchored in the process of socializa- tion and is conditioned by culture and history.	Lytton and Romney, (1991); Rudman and Fairchild (2004); Rudman & Phelan (2008); Vandello & Bosson (2013).

Studies show that gender stereotypes also exist in modern Russia (including within the professional sphere) and that these stereotypes have specific cultural features (Mararitsa, Kazantseva, & Gurieva, 2019; Uryvaev, 2018). According to the Global Gender Gap Report (WEF, 2021), in 2021 Russia ranked 81^st^ among 156 countries on gender parity, that is 32 positions lower than 15 years ago. At the moment, Russia is performing well in education and health equality, ranking 1^st^, with the largest gap observed in the domains of politics and economic participation / career opportunities. Russian social policy is currently characterized by domestic researchers as pro-natalist, aimed at solving demographic problems (Ryabova & Ovcharova, 2016). Ideas regarding the “natural” mission of the sexes and conservative ideas about male and female roles are making a comeback in Russia (Voronina, 2013), configuring gender relations as rooted in power and subordination. Since the situation has recently worsened for Russia, the research described in this study is of high relevance.

Works that address the problem of gender inequality in the professional domain mostly describe career building by female leaders and social-role mechanisms that maintain inequality in organizations (Mararitsa et al., 2019). Most of the research is conducted from the role approach perspective and does not employ a poly-theoretical approach. We were interested in how social representations of gender differences influence the formation of gender inequality in various spheres of life. In fact, we investigated the subjective dimension of gender inequality. In order to study social perceptions of gender differences, the questionnaire was developed and probed. We identified four areas of life in which gender inequalities mostly manifest themselves: family, professional, politics and everyday interaction. Each scale of the questionnaire had its own theoretical foundation, since different spheres of social life were explained by specific theoretical approaches or conceptions, for example, professional issues — by structural (“glass”) phenomena (Folke & Rickne, 2016), family interaction — by the gender-role approach (Eagly & Wood, 1991), everyday interaction — by the phenomenon of “doing gender” (West & Zimmerman, 1987) and gender stereotypes in interpersonal interaction (Stewart et al., 2021).

Based on contemporary research, it is believed that individuals differ a lot in their perceptions of gender inequality ranging from gender blindness, in other words, not taking gender aspects under consideration when it is relevant (Verdonk et al., 2009), to gender awareness, that is awareness of gender-based discrimination and sensitivity to such cases (Morrison, Bourke & Kelley, 2005). The possible correlations of such variability remain under-investigated. It could be a kind of cognitive bias: information that women’s social status is getting better inspires women with an illusion of getting rid of gender prejudice (Spoor & Schmitt, 2011). One could hypothesize that life experience may account for gender awareness, since it grows with age (Neff, Cooper & Woodruff, 2007). Meanwhile, women that have experienced gender discrimination themselves tend to show reluctance to recognize and articulate these events as discrimination (Morrison, Bourke & Kelley, 2005).

The data regarding the positive and negative effects of both polarities in perceptions of gender inequality seem contradictory. Some studies show gender blindness was related to actions — such as risk-taking and negotiation — necessary for reducing gender disparities (Martin & Phillips, 2017), others claim that only recognition of gender differentiation may help to combat gender inequality (Morrison, Bourke & Kelley, 2005).

Driven by inconsistency of empirical studies, the presented research was conducted with an objective to study social perceptions of gender differences in relation to the subjective significance of the gender inequality issue (which includes 4 criteria: gender awareness, frequency of gender inequality witnessing, personal experience of gender discrimination and emotional significance of the experience).

Research question: How does experience and perceptions of gender inequality manifest itself in the content of social perceptions of gender differences? We have focused on the following issues in particular:

*Hypothesis 1.* Social perceptions of gender differences in various spheres are interrelated with the subjective significance of the gender inequality issue.

*Hypothesis 2.* People who articulate their personal experience of gender inequality more readily agree with the idea that gender differences exist in the professional/ career opportunities sphere.

## Methods

### Participants

106 people aged 18 to 68 years participated in the study (M = 30.2, σ = 10.5). The respondents were men (54) and women (52) living in megacities (St. Petersburg and Moscow). Most of our respondents had a full-time day job (as they mentioned in the survey). The absence of statistically significant differences between the samples of men and women in terms of demographic characteristics allowed further comparative analysis.

### Procedure

The study was conducted through an online survey in February–September 2019. The sample was formed by the “snowball” technique within social networks. The time taken to complete the questionnaires was 25–35 minutes. The survey was anonymous.

### Measures

“Social perceptions of gender differences” questionnaire.

The questionnaire consisted of two parts and each part was comprised of four scales. There were two versions of the measure. The first version contained 38 items formulated according to the criteria of gender inequality in various spheres of life. After an expert’s evaluation, 10 items with duplicating content were eliminated, and the wording of the remaining items was corrected. In the 2019 survey, this revised 28-item questionnaire was tested. Respondents were asked to evaluate their agreement with the items on a 5-point scale, where 1 signified “absolutely disagree” and 5 “absolutely agree”. Both direct and reverse items were used, where a greater degree of agreement meant less agreement with gender differences. When calculating the resulting score, the points of reverse items were converted.

The first part of the questionnaire (items 1-20) included four scales concerning gender relations in four spheres of life. We have conditionally named the scales “Family” (*e.g.* “The main task of a woman is housekeeping and caring for her husband and children”), “Work” (*e.g.* “It is more difficult for a woman to stay in a managerial position than for a man”), “Politics” (*e.g.* “A woman is able to succeed in politics”) and “Everyday life” (*e.g.* “Men are better drivers than women”). The second part of the questionnaire (items 21–28) aimed to define social perceptions of gender inequality in an organization where the respondent currently works, according to the criteria of the organizational “glass ceiling” (Folke & Rickne, 2016). The second part also included four scales: “Conditional Vertical Inequality” (*e.g.* “There are more male leaders than female leaders in organizations”), “Bottom-to-top Inequality Acceleration” (*e.g.* “In business, there are far more female middle managers than female top managers”), “Career Advancement Inequality” (*e.g.* “A man moves up the career ladder faster than a woman of the same professional level”), “Diverging Career Trajectories” (*e.g.* “The career paths of women and men in business differ”). For the scales of the first part of the questionnaire (“Work”, “Family”, “Politics”, “Everyday life”) the minimum possible score was 5 points and the maximum possible score was 20 points. For the scales of the second part of the questionnaire, the minimum possible score was 2 points and the maximum possible was 10 points. The higher the score, the more pronounced the perceptions of gender differences in a particular area.

### “Gender Inequality” Questionnaire.

The questionnaire included four direct questions aimed at identifying the respondents’ perceptions of gender inequality and describing their life experiences related to this phenomenon. The questionnaire was also used to identify the degree of subjective significance of the gender inequality issue. The questionnaire included the following questions:

1. Free description of manifestations of gender inequality: “In your opinion, what manifestations/confirmations of gender inequality are there in society? Please list them.” Received responses were classified according to the four categories considered in the first part of the “Social perceptions of gender differences” questionnaire, and the number of mentioned situations was used as a measure of gender awareness.
2. A question about the frequency of observation of such situations: “How often have you observed and heard about such situations?” The question had possible answers rated on a 5-point scale: “never”, “very rarely”, “rarely”, “sometimes”, “often”.
3. A description of the situation of gender inequality in their own life experiences, case studies: “Describe the last unpleasant or offensive situation of manifestation of gender inequality/discrimination that affected you the most.” When coding the data, it was taken into account whether the respondent has described the situation or not; the situations described by the respondents were classified into one of the four categories outlined above (family, work, politics and everyday life).
4. A question aimed to assess the emotional significance of situations of gender inequality: “How upset would you be if you were in this situation?” The question had possible answers rated on a 5-point scale: “not at all”, “a little”, “difficult to answer”, “very”, “extremely”.

Thus, the subjective significance for respondents of the gender inequality issue was assessed by the following 4 indicators used in data processing independently: the number of known manifestations of gender inequality listed by the respondent (gender awareness); the frequency of gender inequality observation (witnessing frequency); the presence or absence of a description of the gender inequality situation in their own life (articulated personal experience) and the degree of unpleasant emotions felt in situations of inequality and discrimination (emotional significance).

Social and Demographic Characteristics.

This questionnaire contained questions about the age, employment, gender, and city of residence of the respondents.

### Mathematical and Statistical Methods of Data Analysis

The assessment of the internal consistency of the scales was carried out using Cronbach’s alpha. When analyzing the reliability of differences between samples, we used the Mann–Whitney U test. Correlation analysis was used to assess the relationships between quantitative variables (indicators of the subjective significance of the gender inequality problem and the degree of agreement with ideas about gender differences in various spheres of life). Spearman’s rank correlation coefficient was used. Factor analysis was employed to analyze the possibility of reducing the eight scales of the questionnaire, which describe ideas about gender differences in various spheres of life, to a smaller number of latent variables. Data analysis was carried out using the SPSS Statistics.

## Results

### Social perceptions of gender differences in various spheres of life

Reliability scores, calculated for the scales included in the “Social perception of gender differences” questionnaire, are presented in *Table 2.* The results of the study confirm the reliability of the following scales: “Work”, “Politics”, “Everyday life”, “Career Advancement Inequality” (Cronbach’s alpha > 0.7 (Furr, 2021; Nasledov, 2011)). Other scales required item corrections (“Family”, “Bottom-to-top Inequality Acceleration”, “Diverging Career Trajectories”; “Conditional Vertical Inequality”).

**Table 2:** Average scores obtained from the questionnaires and the reliability of scales

Scale	Total			Female		Male	
	M	SD	Cronbach’s alpha	M	SD	M	SD
“Work”	11.81	3.74	0.711	13.24	3.62	10.33	3.29
“Family”**	14.65	4.64	0.678	16.78	4.20	12.44	4.04
“Politics”**	7.32	1.87	0.719	7.22	1.60	7.42	2.13
“Everyday life”**	6.81	1.87	0.769	6.46	1.76	7.17	1.93
“Conditional Vertical Inequality”	6.39	2.20	0.526	5.87	1.91	6.92	2.37
“Bottom-to-top Inequality Acceleration”	6.54	2.08	0.605	6.26	1.91	6.83	2.23
“Career Advancement Inequality”*	11.81	3.74	0.841	13.24	3.62	10.33	3.29
“Diverging Career Trajectories”	14.65	4.64	0.690	16.78	4.20	12.44	4.04

Note. * —p < 0.05; ** —p < 0.01, U-test.

Skewness, kurtosis, mean and standard deviation were calculated for each item. This was done in order to test item discrimination of each item and to sift out unfit items. Since the questionnaire is gender-sensitive, descriptive statistics were obtained separately for women and men. This made it possible to identify significant differences in the responses of men and women and meaningfully interpret these differences. According to the indicators of skewness and kurtosis (module of indicators of skewness and kurtosis do not exceed 2 (Furr, 2021; Nasledov, 2011)), the distribution of answers to all the items except item 1 (“A person, regardless of gender, can be successful in any profession”) and item 10 (“A man and a woman should participate equally in the upbringing of children”) can be considered close to normal. The majority of respondents fully agreed that a person, regardless of gender, can be successful in any profession (60.4%) and that a man and a woman should take equal part in the upbringing of children (78.3%).

For each scale of the questionnaire, mean values were calculated both for the sample as a whole and separately for men and women. Differences in the average scores of men and women were assessed using the U-test (*Table 2*).

It can be concluded that the most pronounced spheres where perceptions about gender differences are varied to a greater extent between men and women can be considered the spheres of politics, family and everyday interaction. There is a dissimilarity in the views of men and women about gender differences in career advancement.

In order to study psychological factors of gender inequality and to analyze the possibility of reducing the eight scales of the questionnaire to a smaller number of not-directly-measurable spheres of representation, exploratory factor analysis was performed (*Table 3*).

**Table 3:** Factor loadings of questionnaire scales

Scale	Factor loading	
	1	2
“Work”	.842	–.021
“Family”	–.014	.847
“Politics”	–.091	.816
“Leisure”	.029	.862
“Conditional Vertical Inequality”	.862	.037
“Bottom-to-top Inequality Acceleration”	.866	–.084
“Career Advancement Inequality”	.923	–.145
“Diverging Career Trajectories”	.869	.062

The first factor in the manifestation of the socio-psychological phenomenon of gender inequality is characterized by a high degree of expression of ideas about manifestations of gender inequality in the professional sphere. This can concern career advancement, career trajectories, or vertical gender inequality and its increase in higher positions. We named this factor “Career Inequality”.

The second factor in the manifestation of the phenomenon of gender inequality is characterized by a higher expression of perceptions about gender differences in the family, politics and everyday life. We called the second factor “Differences in Social Spheres”. It implies a comprehensive assessment of the perceptions of gender differences in various social contexts that are not related to employment.

### Social perceptions of gender inequality

When answering the question “In your opinion, what manifestations/confirmations of gender inequality are there in society?” respondents listed 0 to 11 different manifestations, M = 1.79, SD = 2.03. According to the χ^2^-Pearson criterion, men significantly more often did not indicate any manifestation (they gave answers like “I don’t know”, “didn’t notice” or refused to answer) (χ^2^ = 15.63, p < 0.001). Comparative analysis (Mann–Whitney U test) showed that women, on average, described more manifestations of gender inequality known to them (p < 0.001).

We analyzed 190 different manifestations of gender inequality in society described by the respondents and divided them into several categories (*Table 4*). The first four categories corresponded to the four areas of life that we identified when creating the questionnaire “Social perceptions of gender differences”: traditional distribution of roles in family, gender inequality in the professional sphere, gender inequality in politics, and gender inequality and sexism in everyday life. The category “General descriptions of inequality” included descriptions of inequality and sexism that were not related to any specific sphere. The category “Other” included obscure or ambiguous descriptions that could not be assigned to any category without further clarification.

**Table 4:** Manifestations of gender inequality in the perceptions of respondents

Category	Example	quency Fre-	Percent
Family	“Family life, ideas about the duties of a woman”, “men take less part in raising children”, “a clear distribution of roles: a woman gives birth and cleans, a man earns money”.	25	13.2
Professional sphere	“Unequal pay”, “women find it harder to succeed in their careers”, “employers’ fear of hiring young married women.”	48	25.2
Politics	“List of professions prohibited for women”, “different retirement ages”, “attitude towards women in politics”.	32	16.8
Everyday life	“Men often criticize female drivers”, “shaking hands only with men”, “different standards of beauty”, “domestic violence”.	48	25.3
General descriptions of inequality	“Stereotypes”, “attitude towards male as the norm, and female as a deviation from it”, “sexism”.	27	14.2
Other	“Prostitution”, “religion”, “ignorance”, “Rallies, processions of LGBT”.	10	5.3

The frequency of observed situations of gender inequality is presented in *Table 5.*

**Table 5:** Frequency of encountering situations of gender inequality

Option	Total (%)	Male (%)	Female (%)
Often	40.6	20.4	61.5
Sometimes	33.0	42.6	23.1
Rarely	6.6	7.4	5.8
Very rarely	6.6	9.3	3.8
Never	13.2	20.4	5.8

If we consider the response options as an ordinal scale (from 0 — “never” to 4 — “often”), then according to the Mann-Whitney U-test, the women in the study experienced manifestations of gender inequality more often than the men (p < 0.001).

The experienced situation was described by 54.7% of respondents (73.1% of women and 35.7% of men), the rest refused to answer or answered that they did not remember / did not notice such situations. In some cases, respondents described situations that affected their acquaintances, and we considered these situations in the same way as those that happened to the respondent personally, since we were interested in all situations that the respondents considered as a part of their experience.

Men described a total of 20 situations (*Table 6*). We were not able to classify 3 situations into any of the four categories without clarification (these were placed in the category “Other”). Men most often described situations related to gender-based injustice and the pressure of male gender norms and did not describe a single situation regarding family interaction. Women described 38 situations (*Table 6*). Four situations were not classified in any of the four categories considered (“Other”). Women most often described situations of gender inequality related to the performance of traditional female roles in the family, situations of inequality in the professional sphere and manifestations of sexism in everyday life. Women did not describe a single situation regarding inequality in politics.

**Table 6:** Situations of gender inequality in respondents` experience

Category	Example	Frequency	Percent
Male			
Professional sphere	“Some colleagues think that women are not good at their jobs”. “I was falsely accused of harassment at work”.	4	20.0
Politics	“Unequal increase in the retirement age for men and women”.	2	10.0
Everyday life	“In public transport, all seats are occupied by women, men ride standing up”. “People are surprised that I like pink clothes (like sweatshirts) even though I’m a man”.	11	55.0
Other	“Condemnation of 8 years for a young man”. “The cleaner kicked everyone out of the office”.	3	15.0
Female			
Family	“Every time a friend’s mom makes her do something around the house, saying that her father can’t do it, because he is a man, his job is to sit on the couch and watch TV, but women should flutter around him”	8	21.0
Professional sphere	“A colleague was not hired for a managerial position because she could become pregnant and go on maternity leave. Unreliable” “Former boss asked me to write a letter of resignation instead of issuing a formal decree when I was pregnant”	8	21.0
Everyday life	“Woman forced man to give up his seat on the subway and hit him with a bag” “I was told that I should grow my hair to be feminine”	18	47.5
Other	“Imposed repairs, crushing material advantage and selfishness”	4	10.5

For both men and women, situations of gender inequality were most prominently encountered in everyday life. However, for women, manifestations of gender inequality such as the performance of a traditional female role in the family and situations of gender inequality in the professional sphere were also relevant.

The emotional significance of situations of gender inequality was assessed by the answers to the question “How upset would you be if you were in this situation?” (*Table 7*).

**Table 7:** Emotional significance of situations of gender inequality

Option	Total (%)	Female (%)	Male (%)
Extremely	15.1	23.1	7.4
Very	26.4	26.9	25.9
A little	32.1	32.7	31.5
Not at all	17.9	9.6	25.9
Difficult to answer	8.5	7.7	9.3

High emotional significance of situations of manifestation of gender inequality was found in 50.0% of women and 33.3% of men.

Social perceptions of gender differences and the subjective significance of the gender inequality issue

The first hypothesis was that social perceptions of gender differences in various spheres are interrelated with the subjective significance of the gender inequality issue.

The number of known manifestations of gender inequality listed by the respondents (gender awareness) was positively correlated with the “Career Inequality” factor (scale “Work” (r = 0.364, p < 0.001) and scales of the second part of the questionnaire: “Conditional Vertical Inequality” (r = 0.210, p = 0.024), “Bottom-to-top Inequality Acceleration” (r = 0.229, p = 0.018), “Career Advancement Inequality” (r = 0.251, p = 0.009), “Diverging Career Trajectories” (r = 0.216, p = 0.026)); and negatively correlated with the “Differences in Social Spheres” factor (scales: “Family” (r = –0.425, p < 0.001), “Politics” (r = –0.470, p < 0.001), “Everyday life” (r = –0.406, p < 0.001)).

Similar interrelations have been found for the frequency of witnessing gender inequality situations and the emotional significance of such situations.

Consistently, the frequency of observations of gender inequality situations was positively correlated with the factor of “Career Inequality” (scales: “Work” (r = 0,286, p = 0,003), “Bottom-to-top Inequality Acceleration” (r = 0, 295, p = 0.002), “ Career Advancement Inequality” (r = 0.309, p = 0.001)) and negatively correlated with the factor of “Differences in Social Spheres” (scales: “Family” (r = –0.397, p < 0.001), “Politics” (r = –0.362, p < 0.001), “Everyday life” (r = –0.370, p < 0.001)).

The emotional importance of gender inequality situations was positively correlated with the factor of “Career Inequality” (scales: “Work” (r = 0.305, p = 0.002), “Conditional Vertical Inequality” (r = 0.284, p = 0.005), “Bottom-to-top Inequality Acceleration” (r = 0.331, p = 0.001), “Career Advancement Inequality” (r = 0.327, p = 0.001)); and negatively correlated with the factor of “Differences in Social Spheres” (scales “Family” (r = –0.371, p < 0.001), “Politics” (r = –0.227, p = 0.025), “Everyday life” (r = –0.284, p = 0.005)).

Perceptions of gender differences in respondents with and without personal experience of gender inequality

According to the second hypothesis, we expected that people who articulate their personal experience of gender inequality more readily agree with the idea of gender differences in the professional career opportunities.

Using the U-test, significant differences were found in the responses of respondents who described the situation of gender inequality in their own experience and those who did not, the results are shown in *Table 8.* They showed distinctions in perceived degree of gender differences on the factor of “Career Inequality” (scales: “Work” (p = 0.001), “Conditional Vertical Inequality” (p = 0.017), “Bottom-to-top Inequality Acceleration” (p = 0.048), “Career Advancement Inequality”, (p = 0.004), “Diverging Career Trajectories” (p = 0.009)). People with personal experience of inequality scored higher on perceptions of large differences in career opportunities for men and women compared to people who did not mention such experiences (*Table 8*)*.*

**Table 8:** Average scores obtained on scales for respondents who described and did not describe the situation of gender inequality in their experience

Scale	Respondents articulated an experience of gender inequality (n = 58)		Respondents did not articulate an experience of gender inequality (n = 48)	
	M	SD	M	SD
Factor 1 “Career Inequality”				
Work**	14.55	4.18	11,98	3.55
Conditional Vertical Inequality *	7.72	1.84	6.80	1.80
Bottom-to-top Inequality Accelera- tion*	7.10	2.06	6.43	1.53
Career Advancement Inequality**	6.97	2.29	5.63	1.85
Factor 2 “Differences in Social Spheres”				
Family*	10.23	3.84	11.87	3.73
Politics	11.35	3.91	12.35	3.49
Everyday life	14.25	4.86	15.17	4.34

Note. * —p < 0.05; ** —p < 0.01; U-test.

Personal experience of inequality also affects factor 2 “Differences in Social Spheres”, but only in the family relationships domain, where people with personal experience of inequality scored significantly lower compared to those who did not mention such experiences (p = 0.025) (*Table 8).*

## Discussion

The first hypothesis tested was whether social perceptions of gender differences in various spheres would be related to the subjective significance of the gender inequality issue. To this end, we developed two questionnaires within the framework of a poly-theoretical approach: one measure for assessing social perceptions of gender differences in various social domains (family life, professional sphere, politics and everyday interaction) and the other for assessing the subjective significance of the gender inequality issue. The concept of the subjective significance of the gender inequality issue was operationalized through four criteria: gender inequality awareness; frequency of witnessing inequality; articulated personal experience and emotional significance (the degree of unpleasant emotions felt in situations of inequality and discrimination).

Two latent factors were identified to reflect two aspects of gender perceptions: perceptions of gender differences regarding career opportunities (“Career Inequality”) and perceptions of the existence of differences related to gender in various social domains (“Differences in Social Spheres”). Interestingly, the most significant differences in the responses of men and women were found in the second factor: the degree of men’s consent to the existence of gender differences in these areas was higher than that of women.

Significant differences between men and women suggest that perceptions that support the idea of essential differences between genders and expect men to exhibit masculine behavior and women to play a feminine role in family interaction, politics and everyday life, are more pronounced in men. Some perceptions among men of whether gender differences exist are contradictory, which could be a sign of a propensity for hidden forms of sexism. The domain of power (i.e., political and leadership positions) was the most sensitive in terms of confrontational perceptions of gender differences. The revealed gender differences may indicate that women’s perceptions are characterized by greater adherence to perceptions of the absence of essential gender differences in various social domains and by sensitivity to gender inequality. This finding is consistent with other studies that have shown greater male adherence to traditional attitudes and beliefs about masculinity and femininity (Kletsina, 2020).

We have obtained data that fully support Hypothesis 1. All indicators of subjective significance of gender issues were found to be intercorrelated with the two main factors of social perceptions of gender differences (“Career Inequality” and “Differences in Social Spheres”), but in opposing ways. Subjective significance of gender had positive correlations to perceptions of gender differentiation within career opportunities, and negative correlations to the perceptions of crucial differences between men and women. These results support the “gender awareness” proponents claiming that the first step in combating inequality is to be aware of gender-based discrimination and be sensitive to such cases (Morrison, Bourke & Kelley, 2005).

The diversity in the understanding of gender differences in everyday life and in te perceptions of family role distribution can contribute to the “justification” of inequality and to exacerbating conflicts in close relationships. Sexist tendencies are formed and maintained in intimate relationships and the belief in the existence of gender differences functions as a way of controlling closeness in interpersonal relationships. Thus, the basic need for security is fulfilled (Fisher & Hammond, 2019).

Hypothesis 2 was formulated to test the expectation that people who articulate their personal experience of gender inequality more readily agree with the idea of gender differences in the professional career opportunities. We used the term “articulating personal experience” while bearing in mind that certain experiences of gender inequality may be of high emotional significance and thus may be denied, rejected, or hidden (Morrison, Bourke & Kelley, 2005). The results of our study show that when assessing their experience of gender inequality, participants tended to give polarized evaluations to the negative emotions felt in such situations (Table 7) with 25.9% of males totally rejecting negative emotions, and 23.1% of females demonstrating high sensitivity to these kinds of situations.

Hypothesis 2 was also supported by the obtained data. Respondents who described their experiences of gender inequality noticed more gender differences in professional opportunities and career growth, and had an egalitarian perspective about gender differences in the family sphere. We can see now that the elaborated questionnaire “Social perceptions of gender differences” assesses not only perceptions of gender differences (traditionalist or egalitarian in the first part of the measure), but also gender inequality awareness (inequality blindness or sensitivity in the second part). The persistence of gender inequality may be due to interconnected social-psychological mechanisms i.e. traditionalist perceptions of gender differences as essential and inevitable, as well as an inability to detect gender inequality (gender inequality blindness).

## Conclusion

It can be concluded that the subjective significance of the gender inequality issue is interrelated with the social perceptions of gender differences in various spheres of life: for people more committed to the idea of gender differences (rather than similarities) within the family, politics, and everyday life, the problem of gender inequality is less significant; at the same time, they are not aware of gender inequality in the professional sphere, especially when it comes to career opportunities.

The described features of social perceptions of gender differences can become the basis for the formation of hypotheses about their role in the reproduction of the phenomenon of gender inequality. It is possible to formulate hypotheses related to the mechanisms of social cognition and the influence of gender socialization on gender perceptions.

The potential zones of gender conflict were revealed to be the sphere of power, politics and leadership in particular, as well as the sphere of confrontation of ideas about gender differences. This suggests that the hierarchy of gender relations enshrined within culture can be a potential target for social programs to reduce the manifestation of gender inequality in various spheres.

## Limitations

A limitation of the study is the complexity of the validation of non-metric data. Several items of the questionnaire “Social perceptions of gender differences” need revision to increase the reliability of scales. Also, it is necessary to take into account the latent factors identified in this questionnaire and collect a larger dataset to perform confirmatory factor analysis. Further verification of the psychometric properties of the measure is expected to be carried out (validity, social desirability etc.).
